# Using indirect protein interactions for the prediction of Gene Ontology functions

**DOI:** 10.1186/1471-2105-8-S4-S8

**Published:** 2007-05-22

**Authors:** Hon Nian Chua, Wing-Kin Sung, Limsoon Wong

**Affiliations:** 1 School of Computing, National University of Singapore, Singapore, 117543, Singapore

## Abstract

**Background:**

Protein-protein interaction has been used to complement traditional sequence homology to elucidate protein function. Most existing approaches only make use of direct interactions to infer function, and some have studied the application of indirect interactions for functional inference but are unable to improve prediction performance. We have previously proposed an approach, FS-Weighted Averaging, which uses topological weighting and level-2 indirect interactions (protein pairs connected via two interactions) for predicting protein function from protein interactions and have found that it yields predictions with superior precision on yeast proteins over existing approaches. Here we study the use of this technique to predict functional annotations from the Gene Ontology for seven genomes: *Saccharomyces cerevisiae*, *Drosophila melanogaster*, *Caenorhabditis elegans*, *Arabidopsis thaliana*, *Rattus norvegicus*, *Mus musculus*, and *Homo sapiens*.

**Results:**

Our analysis shows that protein-protein interactions provide supplementary coverage over sequence homology in the inference of protein function and is definitely a complement to sequence homology. We also find that FS-Weighted Averaging consistently outperforms two classical approaches, Neighbor Counting and Chi-Square, across the seven genomes for all three categories of the Gene Ontology. By randomly adding and removing interactions from the interactions, we find that Weighted Averaging is also rather robust against noisy interaction data.

**Conclusion:**

We have conducted a comprehensive study over seven genomes. We conclude that FS-Weighted Averaging can effectively make use of indirect interactions to make the inference of protein functions from protein interactions more effective. Furthermore, the technique is general enough to work over a variety of genomes.

## Background

Although sequence similarity search has proven useful in many cases, it has fundamental limitations. First, only a fraction of newly discovered sequences have identifiable homologous genes in current databases. Second, the most prominent vertebrate organisms in GenBank have only a fraction of their genomes present in finished sequences. New bioinformatics methods allow inference of protein function using "associative analysis" of functional properties to complement traditional sequence homology-based methods. Associative properties that have been used to infer function not evident from sequence homology include: co-occurrence of proteins in operons or genome context [1-3]; proteins sharing common domains in fusion proteins [4-6]; proteins with similar phylogenetic profiles [7,8]; proteins with correlated gene expression patterns [9]; and so on. Many approaches have also been proposed for utilizing protein-protein interaction data for functional inference [10-18]. A simple but effective approach is to assign a protein with the function that occurs most frequently in its interaction partners [10]. This is further improved in [11], which predicts function based on chi-square statistics instead of frequency. Some approaches apply machine-learning methods such as clustering and support vector machines [12-14]. Others apply global optimization techniques, such as Markov random fields [15,16] and simulated annealing [17], to propagate predictions so that the function of proteins without characterized neighbors can be predicted. Most of these approaches use the observation that a protein often shares functions with proteins that interact with it (i.e., its level-1 neighbors). However, proteins that interact with the same proteins (i.e., level-2 neighbors) may also have a greater likelihood of sharing similar physical or biochemical characteristics with the target protein. In a previous study [18], we investigated interactions between proteins from the *Saccharomyces cerevisiae *(bakers' yeast) genome from the General Repository of Interaction Datasets (GRID) [19]. We observed that there are proteins that do not share any function with their immediate interaction partners (i.e., level-1 neighbors, S_1_) and yet share some function similarity with the interaction partners of their immediate partners (i.e., level-2 neighbors, S_2_). Out of 4,162 annotated yeast proteins, only 1,999, or 48.0%, share some function with its level-1 neighbors. Of the remaining proteins, 943, or 22.7% of the annotated proteins, share some similarity with at least one of its level-2 neighbors. Less than 2% of the annotated proteins share functions exclusively with level-1 neighbors.

With the assumption that there is no unobserved interaction or annotation, we proposed *indirect functional association *as a reasonable explanation for this observation [18]. Such an indirect functional association can be considered as an instance of the "guilt by association" principle – the common "property" between the level-2 neighbors and the target protein that is used for deriving the "association" is precisely the set of proteins that they both interact with, namely the level-1 neighbors. It is plausible that two proteins that interact with a common set of proteins have a good likelihood of sharing similar physical or biochemical characteristics, and thus exhibit a common function.

While level-2 neighbors may be used to provide greater coverage during function inference, they contain too many false positives to be useful. In order to reduce the impact of these false positives, we devised a topological weighting measure, *Functional Similarity Weight *(FS-Weight), which can be used to identify both direct and indirect (level-2) neighbors that are more likely to share functions. FS-Weight improves the precision of function inference, while the inclusion of FS-weighted level-2 neighbors improves both sensitivity and precision. A new method, *FS-Weighted Averaging*, which incorporates indirect interactions and FS-Weight, was shown to perform better than existing approaches in predicting protein functions for *S. cerevisiae *from protein interactions [18].

Here we study how *FS-Weighted Averaging *performs in predicting protein functions from the protein-protein interaction maps of seven genomes. We also study how the approach performs on noisy interaction data and on predicted interactions. Finally, we show some examples of novel functions predicted for uncharacterized proteins in the *S. cerevisiae *genome and study the predictions that are biologically significant.

## Results

### Coverage of protein-protein interactions

To appreciate the feasibility of protein-protein interactions for function discovery, we want to find out whether protein-protein interactions provide any additional coverage over sequence homology. We look at two well-studied genomes, *S. cerevisiae *and *D. melanogaster*, and examine: 1) how many known functions can be inferred from other proteins with sequence similarity in the genome; 2) how many more functions can be suggested from interaction partners on top of (1); and 3) how many more functions can be suggested from indirect interaction partners on top of (1) and (2).

Each protein sequence is searched against all protein sequences in the Gene Ontology Database [20] using the Basic Local Alignment Search Tool (BLAST) [21] using a range of varying E-value thresholds between 1e-10 to 1. A higher E-value will translate to less significant sequence similarity and vice versa. Hence, a higher E-value threshold will provide better coverage at the expense of lower precision. Proteins with close homologs (E-Value <= 1e-25) from the homology search are excluded from the analysis. The protein interaction network is then examined to find out the number of additional known annotations that can be suggested from direct and indirect interactions. Figure 2 summarizes the findings of this analysis. We find that protein-protein interactions can provide substantial coverage over annotations that cannot be inferred from sequence homology, especially for *biological process *and *cellular component*. We also observe that indirect interactions can provide significant additional coverage over annotations that cannot be inferred from both sequence homology and direct interactions.

### Effectiveness of FS-Weight

The effectiveness of FS-Weighted Averaging for function prediction depends upon the capability of FS-Weight in assigning higher weights to direct and indirect interactions that involve function sharing. Here we study how well FS-Weight scores reflect function similarity. All direct and indirect interactions are first weighted using FS-Weight. For each unique score, we compute the fraction of interactions with weights higher than or equal to this score that share at least one level-4 GO term. The Pearson's correlation coefficient between FS-Weight score and this computed fraction is then computed. This coefficient indicates how well the FS-Weight score of an interaction correlates to the likelihood of function being shared between the proteins involved. Table 2 summarizes the corresponding Pearson's coefficient for each of the seven genomes and each of the three GO categories. We can see that the coefficient values are significant (>0.7) for most cases, indicating that FS-Weight correlates strongly with the likelihood of function sharing. The correlation is lower for molecular function in the *M. musculus *and *R. norvegicus *genomes, but the value is still positive, indicating weaker correlation. No results are available for the molecular function and the cellular component of *C. elegans *due to limited annotation information.

### Function prediction using FS-Weighted Averaging

Using Neighbor Counting and Chi-Square as a benchmark, we want to study the prediction performance of FS-Weighted Averaging over a variety of different genomes. These genomes vary greatly in the availability of annotations and interaction data, which provides a good setup to study the strengths and limitations of the technique. Ten-fold cross validation is performed on each genome using Neighbor Counting, Chi-Square, and FS-Weighted Averaging. Proteins with known functions are randomly divided into 10 groups. In each run, the annotations for one group of proteins will be hidden, and their functions predicted based on the annotations for the remaining groups and the interaction data. The hidden annotations are not available to any preprocessing steps, including reliability estimation and the weighting of interactions. The predictions from the three methods are validated and compared using the two criteria, precision-recall analysis and receiver operating characteristics, as outlined in the Methods section. Only informative GO terms (see Methods) are used for validation.

#### Precision-recall Analysis

The precision versus recall graphs for the prediction of informative GO terms for each of the seven genomes are presented in Figure 3. Only graphs for the biological process category are presented to maintain clarity. Graphs from the molecular function and cellular component categories are provided in the supplementary data (see Additional File 1). We can see from Figure 3 that FS-Weighted Averaging makes predictions with better precision and recall compared to the two other methods for most of the seven genomes. The precision of FS-Weighted Averaging for *R. norvegicus *is less consistent. However, note that due to the lack of annotation information, the informative terms chosen for three genomes, including *R. norvegicus*, may not provide for statistically strong comparisons (see Methods). For the prediction of informative GO terms from the molecular function and cellular component categories, FS-Weighted Averaging also yields better recall and precision over the two other (see Additional File 1). For these categories, no result is available for *C. elegans *as insufficient annotation information is available. We also observe that the superiority of FS-Weighted Averaging over the two other methods is more significant in the genomes with denser interaction networks (i.e., *S. cerevisiae *and *D. melanogaster*).

#### Receiver operating characteristics

To compare the receiver operating characteristics (ROC) [22] of predictions from the three methods, we compute the number of informative GO terms that can be predicted with an ROC ≥ *k*, 0.1 ≥ *k *≥ 1. The number of GO terms that can be predicted with ROC ≥ *k *is plotted against *k *in Figure 4 using the three methods for the seven genomes. Again, only graphs for GO terms from biological process are shown here. We can see that for most of the genomes, FS-Weighted Averaging makes predictions for more GO terms at each ROC threshold. We again observe that the superiority of FS-Weighted Averaging over the two other methods is more significant in the genomes with denser interaction networks. Graphs for the two other GO categories show similar results.

### Function prediction with predicted protein-protein interactions

One of the main limitations in using protein-protein interactions for function prediction is the availability of interaction data. If we can harness predicted interactions, such as those from the STRING database [23], the use of protein-protein interactions for functional inference becomes potentially more useful. The STRING database contains physical interactions as well as interactions predicted from genomic context, coexpression, and previous knowledge. Here we include the interactions for *S. cerevisiae *from STRING into the existing interaction data from BioGRID to study if any improvement can be made from the use of these predicted interactions. Figure 5 shows the precision-recall and ROC analysis of the predictions made by the three methods using 1) only interactions from BioGRID (50,434 unique interactions); and 2) a combination of BioGRID interactions and predicted interactions from STRING (173,797 unique pairs) for informative GO terms from the biological process category. We find that Neighbor Counting and Chi-Square achieved significant improvement in both precision-recall and ROC analysis with the combined interactions, while the performance of FS-Weighted Averaging remains relatively unchanged. This is due to the fact that the predicted interactions from STRING in fact already include many indirect interactions. The average number of annotated neighbors per annotated protein is nearly 65 (see Table 1), which is much higher than the average direct interaction partner per protein of 5 estimated in [24]. Nonetheless, FS-Weighted Averaging is still able to achieve greater ROC for more informative GO terms with the combined interaction data. One interesting point to note is that FS-Weighted Averaging can already achieve outstanding recall and precision as well as ROC performance using the much smaller BioGRID, which is less than one-third the size of the combined interactions!

### Robustness of FS-Weighted Averaging against noise and missing data

FS-Weighted Averaging is designed to take into account the fact that interaction data can be rather noisy [25]. As mentioned in the Methods section, the FS-Weight measure incorporates two forms of countermeasure against noisy interaction data – estimation of the reliability of experimental sources and topological weight. Here we want to study how well the method can perform against noisy data. To simulate noise in interactions, we take the current interaction data and contaminate it with different forms of synthetic noise. We perform this analysis on the *S. cerevisiae *genome since it has a denser interaction data and more complete functional annotations. To differentiate the effects of different types of noise, we modify the interaction data by 1) adding random interactions and 2) randomly removing interactions. Random additions reflect false positives in experimental sources, while random deletions reflect missing data or detection failures. Real protein-protein interactions are likely to include both forms of noise.

FS-Weighted Averaging is used to predict functions from the biological process category using interactions with noise varying from 10% to 50% of the existing interactions applied. As a comparison, we repeated the predictions using Neighbor Counting. Figure 6 presents graphs that show the number of informative GO terms that can be predicted above various ROC thresholds using the two methods on the various perturbed interaction networks. We find that the performances of both methods are less significantly affected by random additions than by random deletions. Interestingly, we also find that the prediction performance of FS-Weighted Averaging actually improves with random additions, while the performance of Neighbor Counting deteriorates with added noise. However, with random deletions, the performance of FS-Weighted Averaging deteriorates slightly faster than that of Neighbor Counting. Nonetheless, FS-Weighted Averaging performs better than Neighbour Counting in all the experiments. These observations indicate that FS-Weighted Averaging is robust to false positives in the interaction data but is less effective in sparser interaction networks.

### Identifying functions better predicted with indirect neighbors

We want to identify functions that can be better predicted with FS-Weighted Averaging. We compute the ROC scores of predictions made by 1) Neighbor Counting (NC) and 2) FS-Weighted Averaging (WA) for each Level-4 GO term annotated by at least 30 proteins. Due to limited annotation and interaction data, we study only 4 genomes: *S. cerevisiae*, *D. melanogaster*, *H. sapiens*, and *M. musculus*. Figure 7 shows a 2D plot of ROC scores of predictions made by Neighbor Counting versus predictions made by FS-Weighted Averaging for biological process GO terms. Each point on the graph represents a Level-4 GO term. A point above the diagonal line indicates that WA yields a better ROC score, while a point below the diagonal line indicates that NC yields a better ROC score.

For all the four genomes in Figure 4, most points on the graph lie above the diagonal line, indicating that FS-Weighted Averaging performed better than Neighbor Counting for most of these GO terms.

To identify GO terms on which FS-Weighted Averaging can achieve the best relative performance over Neighbor Counting, we first select only level-4 GO terms that appear in at least two of the four genomes. For each term, we define a score that reflects the relative ROC score of FS-Weighted Averaging against Neighbor Counting as follows:

FL2(x)=ROCWA(x)ROCNC(x)
 MathType@MTEF@5@5@+=feaafiart1ev1aaatCvAUfKttLearuWrP9MDH5MBPbIqV92AaeXatLxBI9gBaebbnrfifHhDYfgasaacH8akY=wiFfYdH8Gipec8Eeeu0xXdbba9frFj0=OqFfea0dXdd9vqai=hGuQ8kuc9pgc9s8qqaq=dirpe0xb9q8qiLsFr0=vr0=vr0dc8meaabaqaciaacaGaaeqabaqabeGadaaakeaacqWGgbGrdaWgaaWcbaGaemitaWKaeGOmaidabeaakmaabmaabaGaemiEaGhacaGLOaGaayzkaaGaeyypa0ZaaSaaaeaacqWGsbGucqWGpbWtcqWGdbWqdaWgaaWcbaGaem4vaCLaemyqaeeabeaakmaabmaabaGaemiEaGhacaGLOaGaayzkaaaabaGaemOuaiLaem4ta8Kaem4qam0aaSbaaSqaaiabd6eaojabdoeadbqabaGcdaqadaqaaiabdIha4bGaayjkaiaawMcaaaaaaaa@45CE@

where *ROC*_*WA*_*(x) *is the ROC score of the predictions made by FS-Weighted Averaging; and *ROC*_*NC*_*(x) *is the ROC score of the predictions made by Neighbor Counting.

We select the top five terms from each GO category based on their average F_L2 _in the genomes in which they appear and present them in Table 3.

## Discussion

We have shown that FS-Weighted Averaging consistently outperforms Neighbor Counting and Chi-Square in function prediction for the various genomes. By incorporating interaction reliability, topological weight, and indirect interactions, the method can predict more functions with higher precision in all three categories of the Gene Ontology. It is also reasonably resilient against interaction noise, maintaining consistent prediction performance even when a large number of interactions are randomly added to the interaction data. However, we have also seen that the performance edge of FS-Weighted Averaging is less significant in the genomes with sparse interaction networks and also less significant when the interaction network is made sparser by random deletions. This is due to two factors. First, the number of indirect interactions is much lower for sparser networks due to limited connectivity. Indirect interactions can only involve proteins that interact with at least one other protein, i.e., the proteins in the interaction network. Second, the performance of FS-Weighted Averaging is dependent on the effectiveness of the FS-Weight measure, which is limited when the network is sparse.

### Limitations of FS-Weight

We have seen from Table 2 that FS-Weight remains effective even for the sparser networks. However, while FS-Weight can still identify function-sharing interactions for sparser networks, it will miss more interactions that do share function. The topological weight is computed based on the common interaction neighbors of the network. When the interaction network is very sparse, there is often insufficient information in the local topology for FS-Weight to get a confident estimate on functional similarity between proteins. In such cases, FS-Weight assigns a low weight to the interaction. As such, it may limit the contribution of some function-sharing interactions to the function prediction mechanism in FS-Weighted Averaging. Nonetheless, we can see this as a feature rather than a limitation. When a protein interacts with very few proteins, any form of measure that would assign a high reliability score or high confidence in sharing function without additional evidence would be very susceptible to noise and will not give consistent performance over different datasets.

### Examples of indirect functional association

Here we take a look at two examples that illustrate how indirect interactions can provide functional association that cannot be captured through direct interactions.

#### Indirect functional association of biological process

Figure 8 shows the local interaction neighborhood of a protein, YJR147W. To prevent clutter, level-2 neighbors with FS-Weight < 0.05 are excluded. YJR147W has unknown molecular function, and is involved in pseudohyphal growth. YJR147W interacts with only one protein, YGR121C, which is an ammonium permease that participates in the regulation of pseudohyphal growth. Hence it is not possible to assign YJR147W with the biological process *pseudohyphal growth *from YGR121C. However, if we look beyond direct interactions, we find that there are several other proteins that participate in pseudohyphal growth in the level-2 neighbors of YJR147W. These are shown as green nodes in Figure 8.

#### Indirect functional association of molecular function

In the second example, we look at the local interaction neighbourhood of another protein, YBR264C, which is shown in Figure 9. YBR264C, shown as a red node in the figure, is a GTP binding protein. As there are too many level-2 neighbors, we only show those with FS-weight >= 0.05. While YBR264C interacts with nine proteins, none of these shares its molecular function, *GTPase activity*. On the other hand, a number of level-2 neighbors have this function (shown as green nodes).

Interestingly, the level-2 neighbors and YBR264C form a bipartite graph with four proteins: YGL198W, YNL263C, YNL044W, and YER136W. YGL198W is a Ypt-interacting protein that interacts with Rab GTPases. YNL263C and YNL044W have no known molecular function but are known to be involved in ER to Golgi transport.

YER136W is a GDP dissociation inhibitor that regulates vesicle traffic in secretory pathways by regulating the dissociation of GDP from GTP binding proteins. Two of the four proteins have molecular functions that require interaction with GTPases, while the other two have no known molecular function. It is likely that YNL263C and YNL044W, which have no known molecular function, would have molecular functions that involve interaction with GTPases.

We also notice that YGR172C is the only member on its side of the bipartite graph that does not have the molecular function *GTPase activity*. YGR172C is known to be an integral membrane protein required for the biogenesis of ER-derived COPII transport vesicles and has no known molecular function. It is likely that YGR172C would share the molecular function *GTPase activity *with YBR264C.

### Novel predictions for *S. cerevisiae*

Using FS-Weighted Averaging, we predict GO functions for uncharacterized proteins in the interaction network of *S. cerevisiae*. From these predictions, we select predictions with higher confidence by:

1. Excluding GO terms that are associated with fewer than 30 annotated proteins;

2. Excluding GO terms that have an ROC of less than 0.7 during cross validation;

3. For each remaining GO term, retaining only novel predictions that have a score greater than or equal to at least 70% of annotated proteins with the term.

4. Propagating predictions to include ancestor terms for consistency.

These predictions are publicly available at [26]. We welcome collaborations with experimentalists interested in verifying some of these predictions.

## Conclusion

We have investigated the protein-protein interactions from seven genomes and shown that by incorporating topological weighting and indirect neighbors, FS-Weighted Averaging can predict protein function effectively for all three categories of the Gene Ontology. This result is consistent across the seven genomes, indicating that the approach is robust and likely to be generally applicable. We have also studied the impact of noisy interaction data on the performance of FS-Weighted Averaging and find that it is very robust against random perturbations in the interaction network. The study also reveals that FS-Weighted Averaging displays greater effectiveness for sufficiently dense interaction networks as its weighting mechanism requires sufficient local network information.

## Methods

### Interaction and annotation datasets for multiple genomes

In this study, we will cover several genomes, namely *Saccharomyces cerevisiae*, *Drosophila melanogaster *(fruit fly), *Caenorhabditis elegans *(roundworm), *Arabidopsis thaliana *(mouse-ear cress), *Rattus norvegicus *(Norway rat), *Mus musculus *(house mouse), and *Homo sapiens *(human). Protein-protein interactions for *D. melanogaster*, *C. elegans*, and *S. cerevisiae *are obtained from the latest release (2.0.20) of BioGRID (formerly GRID [19]). Interaction data for *A. thaliana*, *R. norvegicus*, *M. musculus*, and *H. sapiens *are obtained from the Biomolecular Interaction Network Database (BIND) [27]. Predicted protein-protein interactions for *S. cerevisiae *are obtained from the Search Tool for the Retrieval of Interacting Genes/Proteins (STRING) database [23].

As genome-specific function annotation schemes may have inherent biases, we use a unified annotation scheme, the Gene Ontology [20], for function annotations. Gene Ontology (GO) terms are arranged in a hierarchical manner with more general terms at the lower level and more specific terms at the higher level. In this study, we define the GO term "biological process" as level 0, its children terms as level 1, and so on. Annotations follow the *true path *rule – a protein annotated with a GO term is also annotated with all its ancestor terms.

Table 1 shows some statistics of the various interaction datasets. We consider only annotated proteins in our study since our interest is in function inference. As the lower levels in the GO hierarchy can be very general, we refer to a protein as "annotated" if it is being annotated with at least one level-4 GO term. The first column depicts the number of interactions between annotated proteins. The second column shows the number of proteins that are annotated *and *have at least one interaction partner. The third column shows the average number of annotated neighbors per (annotated) protein. We use this as a simple indicator of the density of the interaction network. The *S. cerevisiae *dataset has the densest interaction network followed by *D. melanogaster *and *H. Sapiens *datasets. The *R. norvegicus *and *C. elegans *datasets have sparser interaction networks, with less than one annotated neighbor per annotated protein on the average.

### Direct and indirect interactions

**Figure 1:**
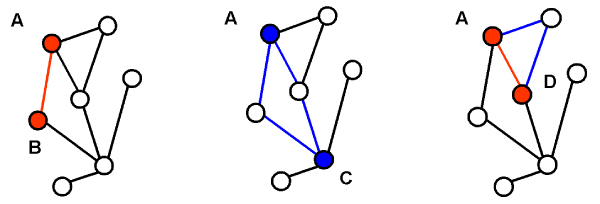
**Direct and indirect interactions**. Nodes represent proteins, while edges represent interactions. Direct interactions between labelled proteins are indicated by red lines, while indirect interactions between labelled proteins are indicated by blue lines.

**Figure 2:**
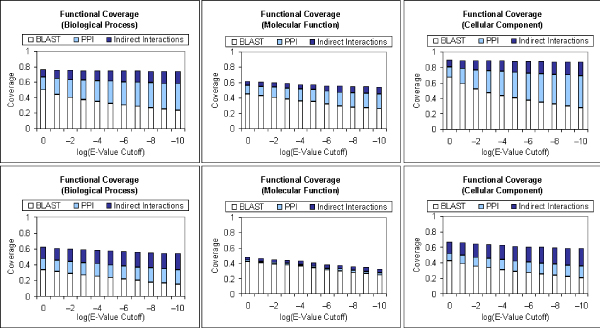
**Functional coverage of protein-protein interactions**. The fraction of known functional annotations that can be suggested through BLAST homology search; and the additional annotations that can be suggested through: 1) direct protein interactions (PPI) and 2) indirect protein interactions. A range of BLAST E-value cutoffs between 1 to 1e-10 is used. BLAST is performed on sequences from the gene ontology database. Proteins with very close homologs (E-value ≤ 1e-25) are excluded from analysis. The top row shows the results from *S. cerevisiae*, and the bottom row shows the results from *D. melanogaster*. The three columns depict results on the biological process (left), molecular function (center) and cellular component (right) categories of the Gene Ontology.

**Figure 3:**
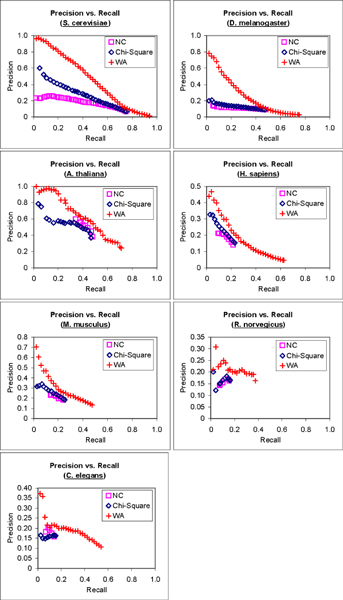
**Precision-recall analysis of predictions by three methods**. Precision vs. recall graphs of the predictions of informative GO terms from the Gene Ontology biological process category using 1) Neighbor Counting (NC); 2) Chi-Square; and 3) FS-Weighted Averaging (WA) for seven genomes.

**Figure 4:**
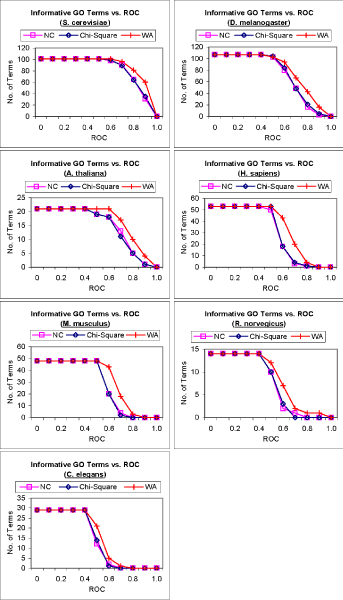
**ROC analysis of predictions by three methods**. Graphs showing the number of informative terms from the Gene Ontology biological process category that can be predicted above or equal various ROC thresholds using 1) Neighbor Counting (NC); 2) Chi-Square; and 3) FS-Weighted Averaging (WA) for seven genomes.

**Figure 5:**
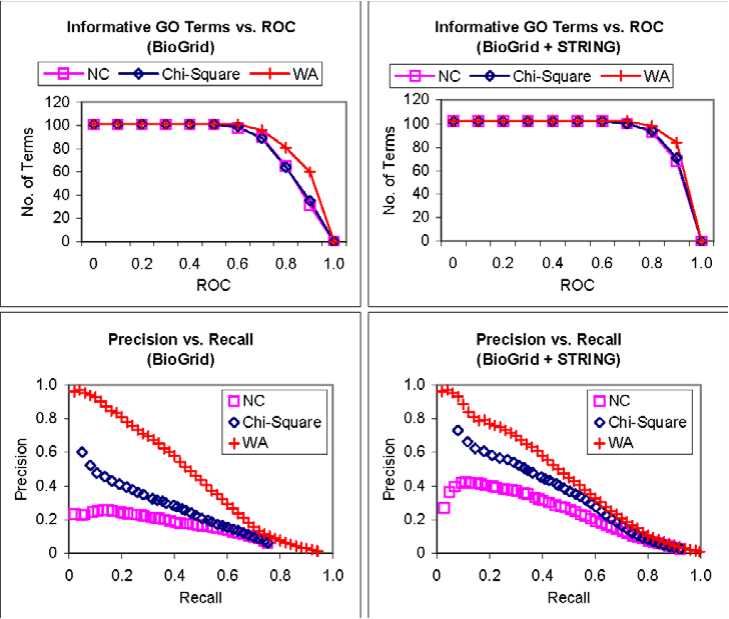
**Incorporating predicted interactions for function prediction**. *Top *– Graphs showing the number of informative terms from the Gene Ontology biological process category that can be predicted greater than or equal to various ROC thresholds for the same methods on BioGRID interactions (left) and a combination of BioGRID interactions and predicted interactions from STRING (right). *Bottom *– Precision vs. recall graphs for predictions of informative terms from the Gene Ontology biological process category using 1) Neighbor Counting (NC); 2) Chi-Square; and 3) FS-Weighted Averaging (WA) on BioGRID interactions (left) and a combination of BioGRID interactions and predicted interactions from STRING (right).

**Figure 6:**
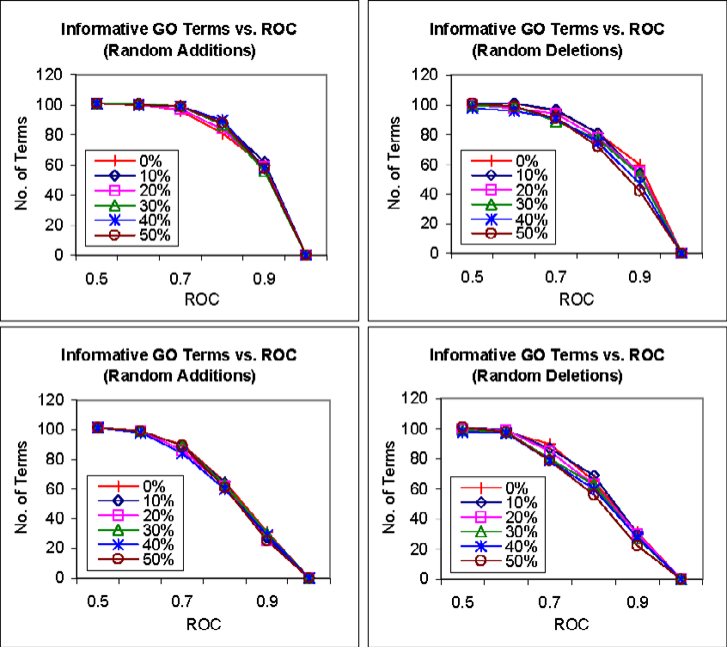
**Effect of noisy interaction data on FS-Weighted Averaging**. Graphs showing the number of informative terms from the Gene Ontology biological process category that can be predicted greater than or equal various ROC thresholds using FS-Weighted Averaging (top) and Neighbor Counting (bottom) on synthetically modified interaction data. Interactions are randomly 1) added to the interaction network (left) and 2) removed from the interaction network (right) in varying degrees from 10% to 50% of the number of interactions in the original interaction.

**Figure 7:**
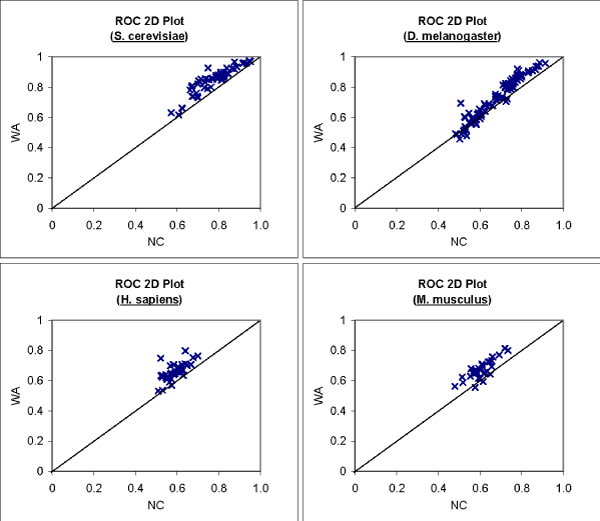
**Effect of indirect interactions on prediction performance for individual GO terms**. 2D Plot of ROC scores of predictions made by neighbor counting versus FS-Weighted Averaging for Level-4 biological process GO terms that are annotated to at least 30 proteins.

**Figure 8:**
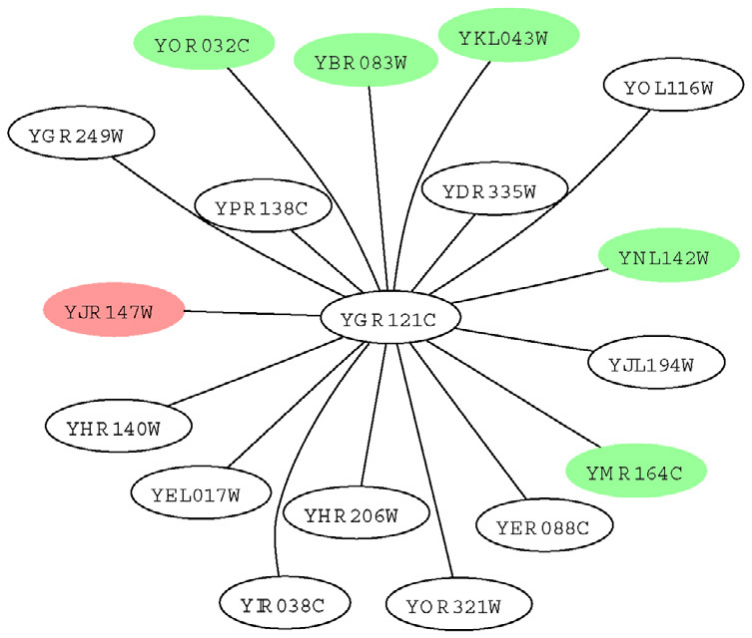
**Example of indirect functional association of biological process**. Graph depicting the local interaction neighborhood of protein YJR147W (shown in red). Proteins shown as green nodes share the biological process *pseudohyphal growth *with YJR147W.

**Figure 9:**
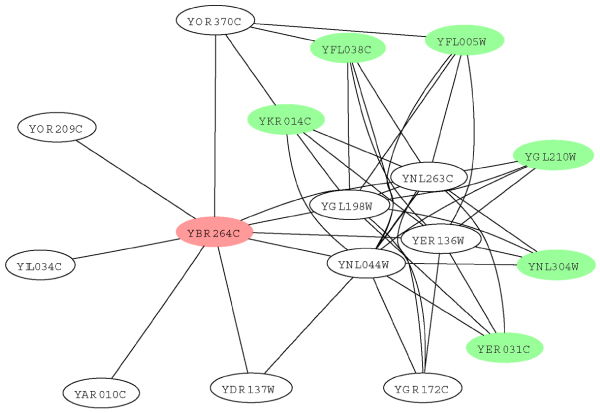
**Example of indirect functional association of molecular function**. Graph depicting the local interaction neighbourhood of protein YBR264C (shown in red). Proteins shown as green node shares the molecular function *GTPase activity* with YBR264C.

We define a direct interaction as an actual interaction between proteins in the protein-protein interaction data. In Figure 1, nodes in the graph represent proteins while edges between the nodes represent protein-protein interactions. There is a direct interaction between proteins A and B. We define an indirect interaction as the sharing of common interaction partners between two proteins (i.e., the two proteins are level-2 neighbors). Figure 1 shows an indirect interaction between proteins A and C. A pair of proteins may have both direct and indirect interactions, as illustrated by proteins A and D in Figure 1. Level-2 neighbors are able to bind to similar proteins; thus they have a higher likelihood of having similar molecular functions. Since subcellular localization shows substantial correlation with molecular function [28], level-2 neighbors are also likely to reside in similar cellular components.

### Topological weighting

Not all indirect interactions indicate function sharing. Indirect relationships are defined upon direct ones and are subjected to noise in the interaction network. Also, while two proteins can interact with a common protein, they may not bind to the common protein at the same site. To identify which indirect interactions are more likely to share functions, we proposed a topological weighting scheme, FS-Weight [18].

The FS-Weight of the direct or indirect interaction between two proteins *u *and *v *is defined as:

SR(u,v)=2∑w∈(N(u)∩N(v))ru,wrv,w(∑w∈N(u)ru,w+∑w∈(N(u)∩N(v))ru,w(1−rv,w))+2∑w∈(N(u)∩N(v))ru,wrv,w+λu,v×2∑w∈(N(u)∩N(v))ru,wrv,w(∑w∈N(v)rv,w+∑w∈(N(u)∩N(v))rv,w(1−ru,w))+2∑w∈(N(u)∩N(v))ru,wrv,w+λv,u
 MathType@MTEF@5@5@+=feaafiart1ev1aaatCvAUfKttLearuWrP9MDH5MBPbIqV92AaeXatLxBI9gBaebbnrfifHhDYfgasaacH8akY=wiFfYdH8Gipec8Eeeu0xXdbba9frFj0=OqFfea0dXdd9vqai=hGuQ8kuc9pgc9s8qqaq=dirpe0xb9q8qiLsFr0=vr0=vr0dc8meaabaqaciaacaGaaeqabaqabeGadaaakqaabeqaaiabdofatnaaBaaaleaacqWGsbGuaeqaaOWaaeWaaeaacqWG1bqDcqGGSaalcqWG2bGDaiaawIcacaGLPaaacqGH9aqpaeaadaWcaaqaaiabikdaYmaaqafabaGaemOCai3aaSbaaSqaaiabdwha1jabcYcaSiabdEha3bqabaGccqWGYbGCdaWgaaWcbaGaemODayNaeiilaWIaem4DaChabeaaaeaacqWG3bWDcqGHiiIZdaqadaqaaiabd6eaonaabmaabaGaemyDauhacaGLOaGaayzkaaGaeyykICSaemOta40aaeWaaeaacqWG2bGDaiaawIcacaGLPaaaaiaawIcacaGLPaaaaeqaniabggHiLdaakeaadaqadaqaamaaqafabaGaemOCai3aaSbaaSqaaiabdwha1jabcYcaSiabdEha3bqabaaabaGaem4DaCNaeyicI4SaemOta40aaeWaaeaacqWG1bqDaiaawIcacaGLPaaaaeqaniabggHiLdGccqGHRaWkdaaeqbqaaiabdkhaYnaaBaaaleaacqWG1bqDcqGGSaalcqWG3bWDaeqaaOWaaeWaaeaacqaIXaqmcqGHsislcqWGYbGCdaWgaaWcbaGaemODayNaeiilaWIaem4DaChabeaaaOGaayjkaiaawMcaaaWcbaGaem4DaCNaeyicI48aaeWaaeaacqWGobGtdaqadaqaaiabdwha1bGaayjkaiaawMcaaiabgMIihlabd6eaonaabmaabaGaemODayhacaGLOaGaayzkaaaacaGLOaGaayzkaaaabeqdcqGHris5aaGccaGLOaGaayzkaaGaey4kaSIaeGOmaiZaaabuaeaacqWGYbGCdaWgaaWcbaGaemyDauNaeiilaWIaem4DaChabeaakiabdkhaYnaaBaaaleaacqWG2bGDcqGGSaalcqWG3bWDaeqaaaqaaiabdEha3jabgIGiopaabmaabaGaemOta40aaeWaaeaacqWG1bqDaiaawIcacaGLPaaacqGHPiYXcqWGobGtdaqadaqaaiabdAha2bGaayjkaiaawMcaaaGaayjkaiaawMcaaaqab0GaeyyeIuoakiabgUcaRGGaciab=T7aSnaaBaaaleaacqWG1bqDcqGGSaalcqWG2bGDaeqaaaaakiabgEna0cqaamaalaaabaGaeGOmaiZaaabuaeaacqWGYbGCdaWgaaWcbaGaemyDauNaeiilaWIaem4DaChabeaakiabdkhaYnaaBaaaleaacqWG2bGDcqGGSaalcqWG3bWDaeqaaaqaaiabdEha3jabgIGiopaabmaabaGaemOta40aaeWaaeaacqWG1bqDaiaawIcacaGLPaaacqGHPiYXcqWGobGtdaqadaqaaiabdAha2bGaayjkaiaawMcaaaGaayjkaiaawMcaaaqab0GaeyyeIuoaaOqaamaabmaabaWaaabuaeaacqWGYbGCdaWgaaWcbaGaemODayNaeiilaWIaem4DaChabeaaaeaacqWG3bWDcqGHiiIZcqWGobGtdaqadaqaaiabdAha2bGaayjkaiaawMcaaaqab0GaeyyeIuoakiabgUcaRmaaqafabaGaemOCai3aaSbaaSqaaiabdAha2jabcYcaSiabdEha3bqabaGcdaqadaqaaiabigdaXiabgkHiTiabdkhaYnaaBaaaleaacqWG1bqDcqGGSaalcqWG3bWDaeqaaaGccaGLOaGaayzkaaaaleaacqWG3bWDcqGHiiIZdaqadaqaaiabd6eaonaabmaabaGaemyDauhacaGLOaGaayzkaaGaeyykICSaemOta40aaeWaaeaacqWG2bGDaiaawIcacaGLPaaaaiaawIcacaGLPaaaaeqaniabggHiLdaakiaawIcacaGLPaaacqGHRaWkcqaIYaGmdaaeqbqaaiabdkhaYnaaBaaaleaacqWG1bqDcqGGSaalcqWG3bWDaeqaaOGaemOCai3aaSbaaSqaaiabdAha2jabcYcaSiabdEha3bqabaaabaGaem4DaCNaeyicI48aaeWaaeaacqWGobGtdaqadaqaaiabdwha1bGaayjkaiaawMcaaiabgMIihlabd6eaonaabmaabaGaemODayhacaGLOaGaayzkaaaacaGLOaGaayzkaaaabeqdcqGHris5aOGaey4kaSIae83UdW2aaSbaaSqaaiabdAha2jabcYcaSiabdwha1bqabaaaaaaaaa@17AE@

N(p) refers to the set that contains protein p and its level-1 neighbors;

*λ*_u,v _is a pseudo-count included in the computation to penalize similarity weights between protein pairs when any of the proteins has very few level-1 neighbors.

r_u,w _refers to the estimated reliability of the interaction between u and w:

ru,v=1−∏i∈Eu,v(1−ri)ni,u,v
 MathType@MTEF@5@5@+=feaafiart1ev1aaatCvAUfKttLearuWrP9MDH5MBPbIqV92AaeXatLxBI9gBaebbnrfifHhDYfgasaacH8akY=wiFfYdH8Gipec8Eeeu0xXdbba9frFj0=OqFfea0dXdd9vqai=hGuQ8kuc9pgc9s8qqaq=dirpe0xb9q8qiLsFr0=vr0=vr0dc8meaabaqaciaacaGaaeqabaqabeGadaaakeaacqWGYbGCdaWgaaWcbaGaemyDauNaeiilaWIaemODayhabeaakiabg2da9iabigdaXiabgkHiTmaarafabaGaeiikaGIaeGymaeJaeyOeI0IaemOCai3aaSbaaSqaaiabdMgaPbqabaGccqGGPaqkaSqaaiabdMgaPjabgIGiolabdweafnaaBaaameaacqWG1bqDcqGGSaalcqWG2bGDaeqaaaWcbeqdcqGHpis1aOWaaWbaaSqabeaacqWGUbGBdaWgaaadbaGaemyAaKMaeiilaWIaemyDauNaeiilaWIaemODayhabeaaaaaaaa@4D57@

*r*_*i *_is the estimated reliability of experimental source *i*;

*E*_*u*,*v *_is the set of experimental sources in which interaction between *u *and *v *is observed; and

*n*_*i*,*u*,*v *_is the number of times that interaction between *u *and *v *is observed from experimental source *i*.

FS-Weight addresses the abovementioned problems in two ways. First, the interaction network is weighted using estimated reliability values to reduce the impact of noise. Second, the weight is largely determined by the number of common interaction partners between the two proteins: if the two proteins share many common neighbors, the likelihood of sharing common physical characteristics increases. The number of non-common neighbors is used as a penalizing factor: if any of the two proteins also bind to many other proteins, then the likelihood of sharing common physical characteristics decreases.

### Reliability of experimental sources

To reduce the impact of noise in the interaction network, the reliability of each experimental source of protein interaction data (e.g., two-hybrid, synthetic lethality) is assessed and weighted. Each source may be assigned an estimated reliability weight by experts based on domain knowledge. However, since we do not have domain knowledge, a simple way to do this is to estimate based on known functions as well as the agreement among independent experimental sources. Here we estimate the reliability of each experimental source by the fraction of unique interactions detected by the experimental source in which at least one level-4 Gene Ontology term is shared. This is done using annotated proteins in the training data during cross validation. The reliability of interactions observed in many independent experimental sources will be combined as described in the definition of FS-Weight. We do not use indirect interactions for the estimation of reliability to avoid circular reasoning as the definition of indirect neighbors is dependent upon the reliability.

### Function prediction

The comparison of FS-Weighted Averaging with many existing approaches has been done in [18] on the yeast genome. Here we will study the performance of the approach on various additional genomes using two classical methods, Neighbor Counting and Chi-Square, as a benchmark:

#### Neighbor Counting

The Neighbor Counting method is proposed in [10]. For each protein *u*, each function *x *is ranked based on the frequency of its occurrence in the interaction partners (level-1 neighbors) of *u*. The rank of each function is used as its score for *u*:

fx(u)=rank(∑v∈Nuδ(v,x))
 MathType@MTEF@5@5@+=feaafiart1ev1aaatCvAUfKttLearuWrP9MDH5MBPbIqV92AaeXatLxBI9gBaebbnrfifHhDYfgasaacH8akY=wiFfYdH8Gipec8Eeeu0xXdbba9frFj0=OqFfea0dXdd9vqai=hGuQ8kuc9pgc9s8qqaq=dirpe0xb9q8qiLsFr0=vr0=vr0dc8meaabaqaciaacaGaaeqabaqabeGadaaakeaacqWGMbGzdaWgaaWcbaGaemiEaGhabeaakmaabmaabaGaemyDauhacaGLOaGaayzkaaGaeyypa0JaemOCaiNaemyyaeMaemOBa4Maem4AaS2aaeWaaeaadaaeqbqaaGGaciab=r7aKnaabmaabaGaemODayNaeiilaWIaemiEaGhacaGLOaGaayzkaaaaleaacqWG2bGDcqGHiiIZcqWGobGtdaWgaaadbaGaemyDauhabeaaaSqab0GaeyyeIuoaaOGaayjkaiaawMcaaaaa@49B0@

*δ(v, x) *= *1 *if *v *has function *x*, *0 *otherwise;

*rank(q(x)) *refers to the rank of the function *x *relative to all functions based on *q(x)*.

#### Chi-Square

The Chi-Square method is proposed in [11]. The approach scores each function *f *observed in the neighbors of a protein *u *using the Chi-Square statistic. The statistic measure computes the deviation of the observed occurrence of function *f *in the neighbors of *u *from its expected occurrence. In [11], the function with the largest chi-square value is assigned to *u*. Since we want to assign multiple functions to each protein, we use the rank of each function as its score instead:

fx(u)=rank((∑v∈Nuδ(v,x)−ex)2ex)
 MathType@MTEF@5@5@+=feaafiart1ev1aaatCvAUfKttLearuWrP9MDH5MBPbIqV92AaeXatLxBI9gBaebbnrfifHhDYfgasaacH8akY=wiFfYdH8Gipec8Eeeu0xXdbba9frFj0=OqFfea0dXdd9vqai=hGuQ8kuc9pgc9s8qqaq=dirpe0xb9q8qiLsFr0=vr0=vr0dc8meaabaqaciaacaGaaeqabaqabeGadaaakeaacqWGMbGzdaWgaaWcbaGaemiEaGhabeaakmaabmaabaGaemyDauhacaGLOaGaayzkaaGaeyypa0JaemOCaiNaemyyaeMaemOBa4Maem4AaS2aaeWaaeaadaWcaaqaamaabmaabaWaaabuaeaaiiGacqWF0oazdaqadaqaaiabdAha2jabcYcaSiabdIha4bGaayjkaiaawMcaaiabgkHiTiabdwgaLnaaBaaaleaacqWG4baEaeqaaaqaaiabdAha2jabgIGiolabd6eaonaaBaaameaacqWG1bqDaeqaaaWcbeqdcqGHris5aaGccaGLOaGaayzkaaWaaWbaaSqabeaacqaIYaGmaaaakeaacqWGLbqzdaWgaaWcbaGaemiEaGhabeaaaaaakiaawIcacaGLPaaaaaa@534E@

*e*_*x *_is the expected number of proteins with function *x *among the interaction partners of *u*, computed by multiplying the number of annotated interaction partners of *u *with the frequency of function *x *among annotated proteins in the interaction map

#### FS-Weighted Averaging

Neighbor Counting uses occurrence-based ranking as a score for functions. A score derived for one protein may not reflect similar confidence as the same score derived for another. In [18], we introduced *FS-Weighted Averaging*, which uses a normalized weighted voting approach. The likelihood that a protein *u *has a function *x *is estimated by:

fx(u)=1Z[∑v∈Nu(SFS(u,v)δ(v,x)+∑w∈Nvmax⁡(SFS(u,v)SFS(v,w),SFS(u,w))δ(w,x))]
 MathType@MTEF@5@5@+=feaafiart1ev1aaatCvAUfKttLearuWrP9MDH5MBPbIqV92AaeXatLxBI9gBaebbnrfifHhDYfgasaacH8akY=wiFfYdH8Gipec8Eeeu0xXdbba9frFj0=OqFfea0dXdd9vqai=hGuQ8kuc9pgc9s8qqaq=dirpe0xb9q8qiLsFr0=vr0=vr0dc8meaabaqaciaacaGaaeqabaqabeGadaaakeaacqWGMbGzdaWgaaWcbaGaemiEaGhabeaakmaabmaabaGaemyDauhacaGLOaGaayzkaaGaeyypa0ZaaSaaaeaacqaIXaqmaeaacqWGAbGwaaWaamWaaeaadaaeqbqaamaabmaabaGaem4uam1aaSbaaSqaaiabdAeagjabdofatbqabaGcdaqadaqaaiabdwha1jabcYcaSiabdAha2bGaayjkaiaawMcaaGGaciab=r7aKnaabmaabaGaemODayNaeiilaWIaemiEaGhacaGLOaGaayzkaaGaey4kaSYaaabuaeaacyGGTbqBcqGGHbqycqGG4baEdaqadaqaaiabdofatnaaBaaaleaacqWGgbGrcqWGtbWuaeqaaOWaaeWaaeaacqWG1bqDcqGGSaalcqWG2bGDaiaawIcacaGLPaaacqWGtbWudaWgaaWcbaGaemOrayKaem4uamfabeaakmaabmaabaGaemODayNaeiilaWIaem4DaChacaGLOaGaayzkaaGaeiilaWIaem4uam1aaSbaaSqaaiabdAeagjabdofatbqabaGcdaqadaqaaiabdwha1jabcYcaSiabdEha3bGaayjkaiaawMcaaaGaayjkaiaawMcaaiab=r7aKnaabmaabaGaem4DaCNaeiilaWIaemiEaGhacaGLOaGaayzkaaaaleaacqWG3bWDcqGHiiIZcqWGobGtdaWgaaadbaGaemODayhabeaaaSqab0GaeyyeIuoaaOGaayjkaiaawMcaaaWcbaGaemODayNaeyicI4SaemOta40aaSbaaWqaaiabdwha1bqabaaaleqaniabggHiLdaakiaawUfacaGLDbaaaaa@82B5@

Z is the sum of all weights:

Z=1+∑v∈Nu(SFS(u,v)+∑w∈Nvmax⁡(SFS(u,v)SFS(v,w),SFS(u,w)))
 MathType@MTEF@5@5@+=feaafiart1ev1aaatCvAUfKttLearuWrP9MDH5MBPbIqV92AaeXatLxBI9gBaebbnrfifHhDYfgasaacH8akY=wiFfYdH8Gipec8Eeeu0xXdbba9frFj0=OqFfea0dXdd9vqai=hGuQ8kuc9pgc9s8qqaq=dirpe0xb9q8qiLsFr0=vr0=vr0dc8meaabaqaciaacaGaaeqabaqabeGadaaakeaacqWGAbGwcqGH9aqpcqaIXaqmcqGHRaWkdaaeqbqaamaabmaabaGaem4uam1aaSbaaSqaaiabdAeagjabdofatbqabaGcdaqadaqaaiabdwha1jabcYcaSiabdAha2bGaayjkaiaawMcaaiabgUcaRmaaqafabaGagiyBa0MaeiyyaeMaeiiEaG3aaeWaaeaacqWGtbWudaWgaaWcbaGaemOrayKaem4uamfabeaakmaabmaabaGaemyDauNaeiilaWIaemODayhacaGLOaGaayzkaaGaem4uam1aaSbaaSqaaiabdAeagjabdofatbqabaGcdaqadaqaaiabdAha2jabcYcaSiabdEha3bGaayjkaiaawMcaaiabcYcaSiabdofatnaaBaaaleaacqWGgbGrcqWGtbWuaeqaaOWaaeWaaeaacqWG1bqDcqGGSaalcqWG3bWDaiaawIcacaGLPaaaaiaawIcacaGLPaaaaSqaaiabdEha3jabgIGiolabd6eaonaaBaaameaacqWG2bGDaeqaaaWcbeqdcqGHris5aaGccaGLOaGaayzkaaaaleaacqWG2bGDcqGHiiIZcqWGobGtdaWgaaadbaGaemyDauhabeaaaSqab0GaeyyeIuoaaaa@6D8F@

In the same equation from [18], we added the background frequency of function *x *to the summation of weights in *f*_*x*_(*u*). When the weights of all the interactions in the local neighbourhood of a protein are very low, the background frequency gives an estimate of the score. This is done so that all proteins can be given a prediction. However, as many of the genomes in this study are very sparse, derived interaction weights are very low. As a result, the background frequency will be given excessive weight, which negatively affects predictions results. Hence we exclude the background frequency from FS-Weighted Averaging in this work.

### Prediction performance evaluation

To evaluate the performance of each approach, we use two popular validation methods, precision-recall analysis and receiver operating characteristics, both of which are described below.

#### Precision-recall analysis

The first method is to plot the precision against recall for the predictions made. Precision-recall analysis indicates the overall prediction performance of a prediction method. It also reflects the ability of a method to assign scores to predictions across different GO terms since it does not differentiate between scores assigned for different terms.

Precision=∑iKki∑iKmiRecall=∑iKki∑iKni
 MathType@MTEF@5@5@+=feaafiart1ev1aaatCvAUfKttLearuWrP9MDH5MBPbIqV92AaeXatLxBI9gBaebbnrfifHhDYfgasaacH8akY=wiFfYdH8Gipec8Eeeu0xXdbba9frFj0=OqFfea0dXdd9vqai=hGuQ8kuc9pgc9s8qqaq=dirpe0xb9q8qiLsFr0=vr0=vr0dc8meaabaqaciaacaGaaeqabaqabeGadaaakeaafaqaaaqacaaabaacbaGae8huaaLae8NCaiNae8xzauMae83yamMae8xAaKMae83CamNae8xAaKMae83Ba8Mae8NBa4Maeyypa0ZaaSaaaeaadaaeWaqaaiabdUgaRnaaBaaaleaacqWGPbqAaeqaaaqaaiabdMgaPbqaaiabdUealbqdcqGHris5aaGcbaWaaabmaeaacqWGTbqBdaWgaaWcbaGaemyAaKgabeaaaeaacqWGPbqAaeaacqWGlbWsa0GaeyyeIuoaaaaakeaacqWFsbGucqWFLbqzcqWFJbWycqWFHbqycqWFSbaBcqWFSbaBcqGH9aqpdaWcaaqaamaaqadabaGaem4AaS2aaSbaaSqaaiabdMgaPbqabaaabaGaemyAaKgabaGaem4saSeaniabggHiLdaakeaadaaeWaqaaiabd6gaUnaaBaaaleaacqWGPbqAaeqaaaqaaiabdMgaPbqaaiabdUealbqdcqGHris5aaaaaaaaaa@6039@

*k*_*i *_is the number of functions correctly predicted for protein *i*;

*m*_*i *_is the number of functions predicted for protein *i*; and

*n*_*i *_is the number of functions annotated for protein *i*

#### Receiver operating characteristics

While precision-recall analysis summarizes the overall prediction performance of a prediction method, it does not tell us about the prediction performance for each term. Since it does not differentiate between predictions made for different terms, it also penalizes methods that do not assign scores that reflect prediction confidence uniformly across different terms. Hence we choose to complement precision-recall analysis with another validation method. The Receiver operating characteristics (ROC) [22] score is the area under the curve derived from plotting true positives as a function of false positives. The ROC score is computed for the predictions for each informative GO term and measures how well the term is predicted for proteins. A higher ROC score indicates a better classifier, and the perfect classifier has an ROC score of 1. For any given GO term, if no prediction is made for a protein, we assume that the lowest possible score is assigned. The ROC does not reflect the recall of a method and does not differentiate between a method with very low recall and a method with high recall but low precision. Hence the two validation methods are complementary.

Since statistical measures are used for the validation of predictions, we only consider terms that are annotated to a reasonably large number of proteins to ensure that conclusions drawn from these measures are statistically sound. We adopt the approach of informative functional classes used in [9] and [18]. For each of the 3 GO categories – biological process, molecular function, and cellular component – we define an informative GO term as a term which is annotated to at least *n *proteins and does not have any child term that is annotated to at least *n *proteins. *n *= 30 is used for the *S. cerevisiae*, *D. melanogaster*, *M. musculus*, and *H. sapiens *genomes. For the remaining genomes, *n *= 10 is used instead as there are few or no informative terms for validation when *n *= 30 is used.

## Abbreviations

ER – Endoplasmic Recticulum; FS-Weight – Functional Similarity Weight; GTP – Guanosine triphosphate; GTPases – GTP binding proteins; GDP – Guanosine diphosphate; NC – Neighbour Counting; ROC – Receiver Operating Characteristics; WA – FS-Weighted Averaging

## Competing interests

The authors declare that they have no competing interests.

## Authors' contributions

HNC was responsible for conceptualization and implementation and drafted the manuscript. WKS and LW participated in conceptualization and discussion as well as revision of the draft.

## Supplementary Material

Additional file 1 **Supplementary Text and Figures**. Precision vs. recall and ROC analyses of the predictions of informative GO terms from the Gene Ontology molecular function and cellular component categories using Neighbor Counting (NC), Chi-Square, and FS-Weighted Averaging (WA) for seven genomes. Click here for file

## Figures and Tables

**Table 1:** Statistics of interaction data from seven genomes

Genome	Interactions involving annotated proteins	Annotated Proteins	Avg. no. of annotated neighbors per protein
*S. cerevisiae*	50,434	4,005	21.6654
*D. melanogaster*	24,991	2,763	4.2823
*A. thaliana*	909	382	1.8386
*H. sapiens*	5,784	5,784	1.6761
*M. musculus*	1,892	1,892	1.3595
*R. norvegicus*	590	590	0.9803
*C. elegans*	4,349	382	0.7382
*S. cerevisiae (predicted)*	145,003	3,987	64.5949

**Table 2:** Pearson's coefficient between FS-Weight and function sharing likelihood for each genome and GO category

Genomes	Biological Process	Molecular Function	Cellular Component
*S. cerevisiae*	0.846	0.782	0.858
*D. melanogaster*	0.744	0.817	0.921
*A. thaliana*	0.938	0.872	0.728
*H. sapiens*	0.899	0.813	0.923
*M. musculus*	0.911	0.574	0.890
*R. norvegicus*	0.904	0.423	0.854
*C. elegans*	0.673	-	-

**Table 3:** Level-4 GO terms annotated to at least 30 proteins in at least two genomes with the top five average FL2 scores for each category of the Gene Ontology

GO term	Avg. F_L2 _score
***Biological process***	
Cellular biosynthesis	1.2381
Regulation of kinase activity	1.2159
Regulation of biosynthesis	1.1546
Cellular macromolecule metabolism	1.1407
Response to pest, pathogen, or parasite	1.1367
***Molecular function***	
Phosphotransferase activity, alcohol group as acceptor	1.1758
Transcription factor activity	1.1672
Kinase activity	1.1640
Transcription cofactor activity	1.1639
Calcium ion binding	1.1312
***Cellular component***	
Eukaryotic 48S initiation complex	1.6388
Eukaryotic 43S preinitiation complex	1.4247
Cytosol	1.2630
Intrinsic to plasma membrane	1.1625
Intracellular non-membrane-bound organelle	1.1393
