# Supplementary material for: Using indirect protein interactions for the prediction of Gene Ontology functions
Source: BMC Bioinformatics. 2007 May 22;8(Suppl 4):S8. doi: 10.1186/1471-2105-8-S4-S8 (PMC1892087; doi:10.1186/1471-2105-8-S4-S8)
Supplement: Additional file 1 — Supplementary Text and Figures. Precision vs. recall and ROC analyses of the predictions of informative GO terms from the Gene Ontology molecular function and cellular component categories using Neighbor Counting (NC), Chi-Square, and FS-Weighted Averaging (WA) for seven genomes. [file 1471-2105-8-S4-S8-S1.pdf]

Using Indirect Protein Interactions for the prediction of Gene Ontology functions

Hon Nian Chua, Wing-Kin Sung, Limsoon Wong§

National University of Singapore, School of Computing, 3 Science Drive 2, Singapore 117543

§Corresponding author

Email addresses:

HNC: g0306417@nus.edu.sg

WKS: ksung@comp.nus.edu.sg

LW: wongls@comp.nus.edu.sg

Supplementary Results

Function prediction using FS-Weighted Averaging

Precision–Recall Analysis

Figure S1 shows the precision versus recall graphs for the prediction of informative GO terms for the *molecular function* and *cellular component* categories.

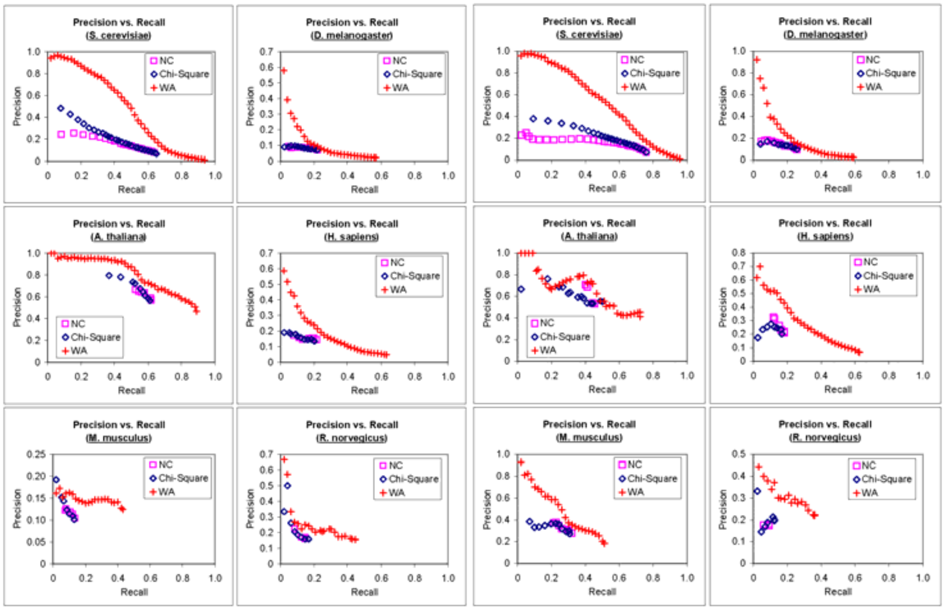

Figure S1 – Precision–Recall Analysis of predictions by three methods

Precision vs. Recall graphs of the predictions of informative GO terms from the Gene Ontology biological process category using 1) *Neighbor Counting (NC)*; 2) *Chi-Square*; and 3) *FS-Weighted Averaging (WA)* for seven genomes.

Left – Graphs for GO terms from the *molecular function* category

Right – Graphs for GO terms from the *cellular component* category

Receiver Operating Characteristics
Figure S2 shows the number of informative GO terms from the *molecular function* and *cellular component* categories that can be predicted equal or above a range of ROC thresholds.

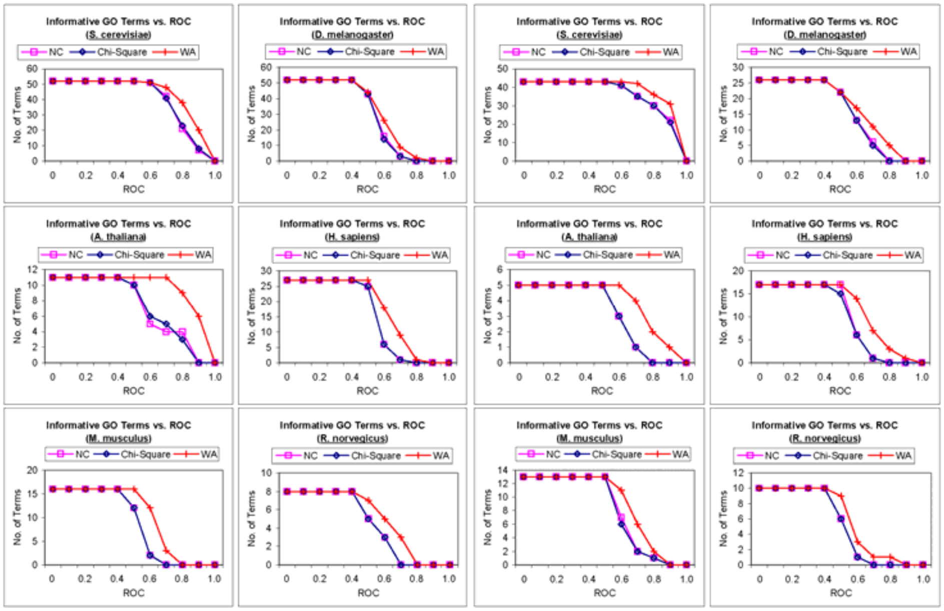

Figure S2 - ROC Analysis of predictions by three methods

Graphs showing the number of informative terms from the Gene Ontology biological process category that can be predicted above or equal various ROC thresholds using 1) *Neighbor Counting (NC)*; 2) *Chi-Square*; and 3) *FS-Weighted Averaging (WA)* for seven genomes.
Left – Graphs for GO terms from the *molecular function* category
Right – Graphs for GO terms from the *cellular component* category
